# The Effect of Modifiers on the Performance of Ni/CeO_2_ and Ni/La_2_O_3_ Catalysts in the Oxy–Steam Reforming of LNG

**DOI:** 10.3390/ijms22169076

**Published:** 2021-08-23

**Authors:** Magdalena Mosinska, Waldemar Maniukiewicz, Malgorzata I. Szynkowska-Jozwik, Pawel Mierczynski

**Affiliations:** Institute of General and Ecological Chemistry, Faculty of Chemistry, Lodz University of Technology, Zeromskiego 116, 90-924 Lodz, Poland; magdalena.mosinska@dokt.p.lodz.pl (M.M.); waldemar.maniukiewicz@p.lodz.pl (W.M.); malgorzata.szynkowska@p.lodz.pl (M.I.S.-J.)

**Keywords:** LNG, reforming of LNG, syngas, nickel catalyst, hydrogen production

## Abstract

This work interrogates for the first time the catalytic properties of various monometallic Ni catalysts in the oxy-steam reforming of LNG. Various research techniques, including X-ray diffraction (XRD), specific surface area and porosity analysis (BET method), scanning electron microscopy with X-ray microanalysis (SEM-EDS), temperature-programmed desorption of ammonia (TPD-NH_3_), temperature-programmed reduction (TPR-H_2_) and the FTIR method, were used to study their physicochemical properties. The mechanism of the oxy-steam reforming of LNG is also discussed in this paper. The high activity of monometallic catalysts supported on 5% La_2_O_3_–CeO_2_ and 5% ZrO_2_–CeO_2_ oxides in the studied process have been proven and explained on the basis of their acidity, specific surface area, sorption properties in relation to the reaction products, the crystallite size of the metallic nickel and their phase composition.

## 1. Introduction

Nowadays, an important issue is the optimal use of natural sources to minimize the human impact on environment. Renewable energy sources are considered to provide clean energy which does not have harmful effects on the environment and human health. Currently, many researchers have focused their attention on hydrogen as an energy carrier [1,2,3,4]. It is the most promising source of energy due to its high purity and the fact that no pollution is emitted during its combustion. The biggest advantage of hydrogen is also its high energy density [5]. As a result of these attributes, hydrogen has found wide applications as a fuel source to power fuel cells for stationary systems and mobile vehicles [6,7,8,9]. Hydrogen can be produced through the catalytic reforming of hydrocarbons. The well-known methods of hydrogen production are partial oxidation, steam or dry reforming and oxidative steam reforming of hydrocarbons [10,11,12,13,14,15,16,17]. Comparing all hydrocarbon reforming methods, methane steam reforming plays a key role in the global energy economy [18,19,20,21,22]. It is well known that the steam reforming of methane leads to the production of syngas with the highest H_2_:CO ratio ≥3 [23,24,25]. The widespread use of LNG pipelines in modern cities has resulted in a focus on using this raw material to produce hydrogen [26]. Liquefied natural gas (LNG) is a mixture of methane (95%) and other components (only 5%) and therefore it can be used in reforming reactions to produce hydrogen [27,28,29,30]. LNG is also easy to store and transport due to the fact that in the liquid phase it occupies about a 600-times-smaller volume than it occupies in the gas phase. In this respect, it is essential to develop and investigate the highly efficient catalytic process of LNG reforming [31,32].

Cerium oxide is the one of the most common catalyst support materials [33,34,35,36]. It is well known that CeO_2_ improves the active metal dispersion and limits the agglomeration of the particles on the catalyst surface. The addition of CeO_2_ can influence the Ni size and enhance the metal–support interaction. The characteristic property of CeO_2_-containing catalysts is their ability to transfer mobile oxygen, which provides oxidation/reduction conditions and resistance to coke deposition [37,38,39]. The scientific literature contains a wide range of reports about the influence of different promoters on the catalytic activity, stability and selectivity of Ni catalysts in hydrocarbon reforming processes [10,12,40,41]. The impact of metal oxide addition on the catalytic properties of catalytic systems depends on the amount of additive introduced, but even the addition of a small amount of metal oxides affects the physicochemical properties, catalytic activity and stability of the catalyst. The addition of rare earth oxides, such as La_2_O_3_, to Ni-based catalysts facilitates the dispersion and reduction of NiO species present on the catalyst surface. This modifier also improves the stability of nickel catalysts by facilitating the oxidation of carbon deposited on the surface, which directly reduces the formation of carbon deposits [42,43,44]. It is also known that the ZrO_2_ modifier of nickel catalysts can also enhance coking resistance and stabilize the structural and chemical properties of the catalysts. The addition of zirconium oxide to Ni-based catalysts increases the nickel dispersion and active phase surface area. It has also been proven that the addition of ZrO_2_ to nickel catalysts neutralizes the acidity of the catalyst surface [45].

The main aim of this work was to determine the catalytic performance and mechanism of the oxy-steam reforming of LNG using nickel catalysts supported on mono-oxides (La_2_O_3_ or CeO_2_) or binary oxides: 5% CeO_2_–La_2_O_3_, 5% ZrO_2_–La_2_O_3_, 5% La_2_O_3_–CeO_2_ and 5% ZrO_2_–CeO_2_. The influence of the addition of lanthanum, zirconium and cerium oxides to CeO_2_ or La_2_O_3_ supports on the physicochemical properties of nickel catalysts were extensively studied in this work. To achieve the intended purposes, the catalysts were characterized using a variety of research techniques, including temperature-programmed reduction of hydrogen (TPR-H_2_), temperature-programmed desorption of ammonia (TPD-NH_3_), X-ray diffraction (XRD), low temperature nitrogen adsorption (BET), Fourier transform infrared spectroscopy (FTIR) and scanning electron microscopy with an energy dispersive spectrometer (SEM-EDS), respectively. The correlation between the physicochemical properties and the reactivity of the investigated catalyst in the studied process was described. In addition, the reaction mechanism of the oxy-steam reforming of LNG was also studied and a description is suggested in this paper.

## 2. Results and Discussion

### 2.1. BET Analysis

**Figure 1:**
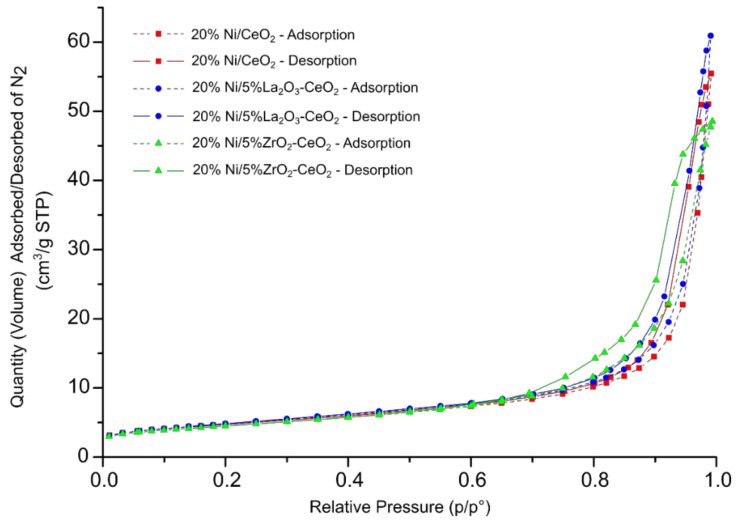
Nitrogen adsorption–desorption isotherms for 20% Ni/CeO_2_, 20% Ni/5% La_2_O_3_–CeO_2_ and 20% Ni/5% ZrO_2_–CeO_2_ catalysts calcined in an air atmosphere at 400 °C for 4 h.

**Figure 2:**
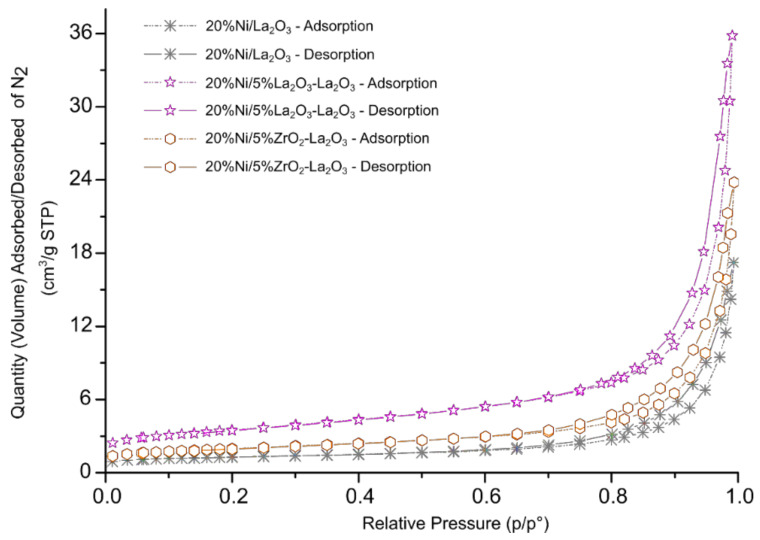
Nitrogen adsorption–desorption isotherms for 20% Ni/La_2_O_3_, 20% Ni/5% CeO_2_–La_2_O_3_ and 20% Ni/5% ZrO_2_–La_2_O_3_ catalysts calcined in an air atmosphere at 400 °C for 4 h.

Specific surface area (SSA) measurements were carried out using the N_2_ adsorption–desorption method to determine the BET surface area, monolayer capacity and average pore radius of the investigated supports and nickel-supported catalysts. The SSA results are given in Table 1. The SSA measurements showed that supports with higher CeO_2_ content exhibited higher specific surface area values in comparison to La_2_O_3_ and modified 5% CeO_2_–La_2_O_3_ (4.91 m^2^/g) and 5% CeO_2_–La_2_O_3_ systems. The lowest SSA value among all the tested carriers was exhibited by La_2_O_3_ oxide (0.44 m^2^/g). The addition of CeO_2_ or ZrO_2_ oxide onto the La_2_O_3_ surface resulted in an increase in the specific surface area. In addition, the obtained results showed that the highest specific surface area among the Ni catalysts was that of the nickel catalyst supported on 5% La_2_O_3_–CeO_2_. The nickel catalyst supported on CeO_2_ oxide showed higher values of SSA (16.69 m^2^/g) and monolayer capacity (0.09 cm^3^/g) compared to the 20% Ni/La_2_O_3_ system. Furthermore, the 20% Ni/La_2_O_3_ catalyst had the lowest BET surface area, monolayer capacity and the highest average pore radius compared to all the studied Ni systems. In the case of the nickel catalyst supported on CeO_2_, the introduction of small amounts of ZrO_2_ and La_2_O_3_ had no significant influence on the SSA results. On the other hand, in the case of the nickel catalyst supported on La_2_O_3_, the introduction of CeO_2_ and ZrO_2_ resulted in an increase in the BET surface area and monolayer capacity, as well as a decrease in the average pore radius in the case of these catalytic systems. The values of the average pore radius for the investigated nickel catalysts are in the range 7.92–12.70 nm. The BET surface area and monolayer capacity of the studied Ni catalysts can be presented in the following order: 20% Ni/La_2_O_3_ > 20% Ni/5% ZrO_2_–La_2_O_3_ > 20% Ni/5% CeO_2_–La_2_O_3_ > 20% Ni/5% ZrO_2_–CeO_2_ > 20% Ni/CeO_2_ > 20% Ni/5% La_2_O_3_–CeO_2_. It is worth mentioning that the last three catalysts with the highest specific surface area values also showed the highest activity in the oxygen-steam reforming of LNG. The N_2_ adsorption–desorption isotherms obtained for the investigated nickel catalysts are presented in Figure 1 and Figure 2, respectively. Brunauer, Emett and Teller classified five types of physical adsorption isotherms, which are called BET isotherms. We can assign all the studied catalyst systems to the second type of isotherms. This type is associated with the formation of a multi-molecular layer. The increase in pressure reduces the number of active sites occupied by one adsorbate molecule due to the fact that double and triple adsorption complexes are formed [46]. De Boera [47] proposed the standard shapes of the hysteresis loop, which correspond to the nature of the pores present on the adsorbent surface.

This knowledge can allow us to better understand the texture of the investigated catalyst surface. The types of hysteresis loops which we can observe in Figure 1 and Figure 2 correspond to the cylindrical shapes of the pores with different cross-sectional shapes (round, triangular and polygonal) which have a similar radius. The results of the SSA measurements correspond to our previous studies [45] of similar nickel catalysts supported on CeO_2_, La_2_O_3_, CeO_2_–La_2_O_3_ (2:1 and 1:2), CeO_2_–ZrO_2_ (2:1) and La_2_O_3_–ZrO_2_ (2:1) systems. We also observed for all investigated catalytic systems the most common type of adsorption isotherms (type II).

### 2.2. The Acidity of the Catalyst Systems Tested (TPD-NH_3_)

**Figure 3:**
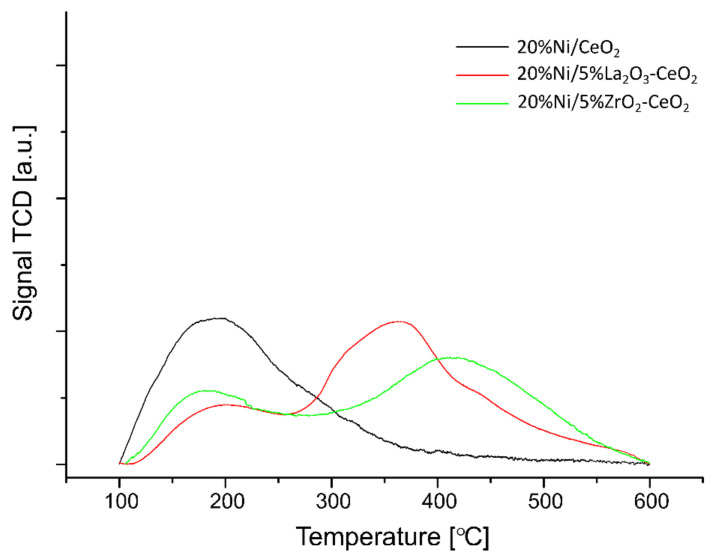
The temperature-programmed desorption of ammonia profiles recorded for 20% Ni/CeO_2_, 20% Ni/5% La_2_O_3_–CeO_2_ and 20% Ni/5% ZrO_2_–CeO_2_ catalysts reduced in hydrogen atmosphere for 1 h at 500 °C.

**Figure 4:**
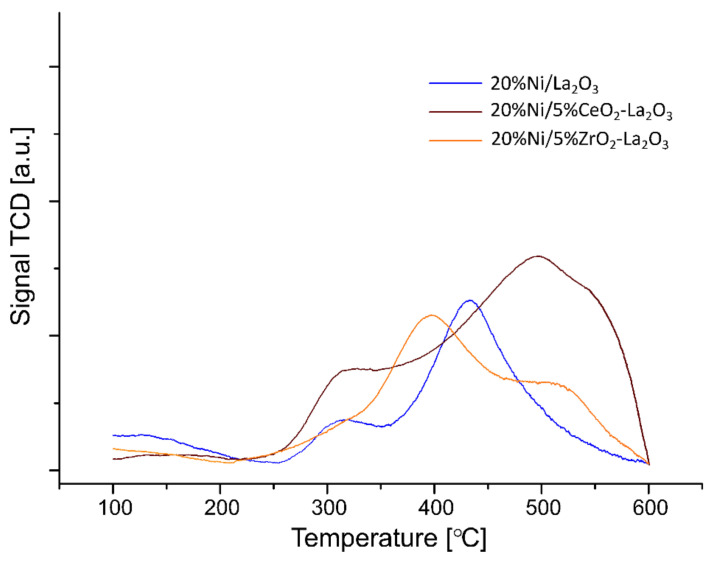
The temperature programmed desorption of ammonia profiles recorded for 20% Ni/La_2_O_3_, 20% Ni/5% CeO_2_–La_2_O_3_ and 20% Ni/5% ZrO_2_–La_2_O_3_ catalysts reduced in hydrogen atmosphere for 1 h at 500 °C.

The temperature-programmed desorption of ammonia measurements (TPD-NH_3_) was performed to determine the total acidity and distribution of the acid centers on the surface of the investigated nickel catalysts. The studied samples were reduced in pure hydrogen at 500 °C for 1 h before each TPD-NH_3_ run. The obtained acidity results are shown in Table 2 and Figure 3 and Figure 4, respectively. The TPD-NH_3_ results confirmed the presence of weak, medium and strong acid centers, detected on the surface of all tested catalyst samples. The 20% Ni/CeO_2_ and 20% Ni/La_2_O_3_ catalysts showed similar total acidity values, equal 0.30 and 0.35 mmol/g, respectively. However, in the case of the 20% Ni/CeO_2_ system, we observed higher values of weak acid centers compared to the 20% Ni/La_2_O_3_ catalyst, which exhibited higher values of medium and strong acid centers on the surface. The addition of ZrO_2_ to the CeO_2_ support surface in the case of the nickel catalyst increased the total acidity. An increase in the amount of medium and the high strength of the acid sites was observed in the case of the 20% Ni/5% ZrO_2_–CeO_2_ system. On the other hand, the nickel catalyst supported on the 5% La_2_O_3_-CeO_2_ carrier showed lower total acidity and an increased amount of medium and strong acid centers on its surface compared to the 20% Ni/CeO_2_ system. The introduction of ZrO_2_ or CeO_2_ to the La_2_O_3_ carrier resulted in an increase in the total acidity of nickel catalyst compared to the 20% Ni/La_2_O_3_ system. In the case of the 20% Ni/5% CeO_2_–La_2_O_3_ catalyst, we also observed the highest value of total acidity (1.69 mmol/g) among all the investigated catalysts and the highest amount of strong acid centers (1.37 mmol/g). It is also worth noting that all catalysts with the addition of a low content of metal oxide (ZrO_2_, CeO_2_) exhibited higher hydrocarbon conversion compared to the non-promoted Ni catalyst. In addition, the Ni/CeO_2_ catalysts promoted by 5% ZrO_2_ or 5% La_2_O_3_ oxides exhibited hydrogen formation even at 500 °C, which was not observed when the process was realized on unpromoted catalyst. These results confirmed that increasing the acidity improves the catalytic activity and hydrogen yield during the process performed using Ni catalysts during the oxy–steam reforming of LNG.

### 2.3. Phase Composition Studies

**Figure 5:**
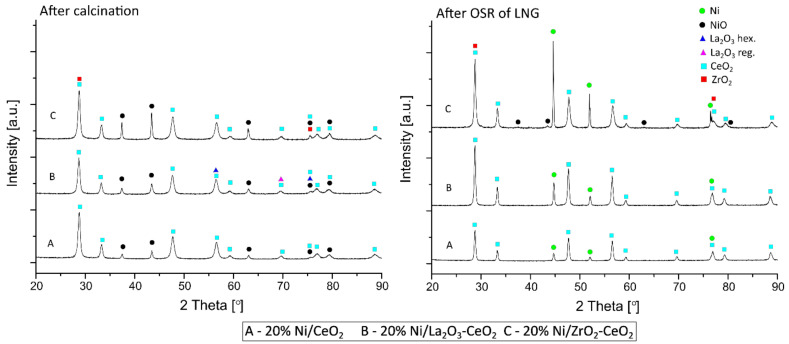
XRD curves of the 20% Ni/CeO_2_, 20% Ni/5% La_2_O_3_–CeO_2_ and 20% Ni/5% ZrO_2_–CeO_2_ catalysts calcined in an air atmosphere for 4 h at 400 °C and after the oxy-steam reforming of LNG.

**Figure 6:**
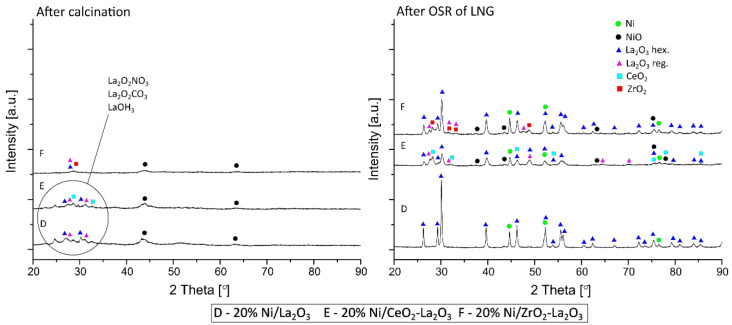
XRD curves of the 20% Ni/La_2_O_3_, 20% Ni/5% CeO_2_–La_2_O_3_ and 20% Ni/5% ZrO_2_–La_2_O_3_ catalysts calcined in an air atmosphere for 4 h at 400 °C and spent in the oxy-steam reforming of LNG.

The phase composition studies were performed using an X-ray diffraction (XRD) technique in order to determine the physicochemical properties of the investigated nickel catalysts and correlate them with their reactivity results in the oxy-steam reforming of LNG. The results of the XRD measurements are given in Figure 5 and Figure 6. The X-ray diffraction curves of the investigated catalytic systems were recorded for catalysts after a calcination process carried out at 400 °C for 4 h in an air atmosphere and after the OSR of LNG reaction, which was performed in the temperature range 400 °C–900 °C. The addition of La_2_O_3_, ZrO_2_ and CeO_2_ into the supported structure did not cause significant changes in their phase composition. Figure 5 presents the diffraction curves recorded for nickel catalysts supported on CeO_2_ (A), 5%La_2_O_3_–CeO_2_ (B) and 5%ZrO_2_–CeO_2_ (C), respectively. In the case of all samples after the calcination process, the XRD curves present the diffraction peaks assigned to NiO (positioned at 2θ angles of 37.3°, 43.2°, 62.8°, 75.5° and 79.3°) and CeO_2_ (positioned at 2θ angles of 28.6°, 33.3°, 47.6°, 56.5°, 59.3°, 69.8°, 75.5°, 77.1°, 79.7° and 88.8°) phases. In addition, the diffraction curves of Ni catalysts with small amounts of La_2_O_3_ or ZrO_2_ oxides showed the presence of diffraction peaks, which can be attributed to the crystallographic phases of La_2_O_3_ hex. (positioned at 2θ angles of 56.5° and 75.5°) and La_2_O_3_ reg. (positioned at the 2θ angle of 69.8°) for the 20% Ni/5% La_2_O_3_–CeO_2_ catalyst and diffraction peaks assigned to the ZrO_2_ phase (positioned at 2θ angles of 28.6° and 75.5°) for the 20% Ni/5% ZrO_2_–CeO_2_ system.

On the other hand, the XRD patterns recorded for spent 20% Ni/CeO_2_, 20% Ni/5% La_2_O_3_–CeO_2_ and 20% Ni/5% ZrO_2_–CeO_2_ catalysts showed the occurrence of the same oxidic phases assigned to the support components. In addition, for all the investigated samples, the peaks assigned to the metallic Ni (positioned at 2θ angles of 44.5°, 51.6° and 75.8°) phase were detected. It is worth mentioning that, for the 20% Ni/5% ZrO_2_–CeO_2_ catalyst, we also observed the diffraction peaks assigned to the nickel oxide phase, which can be explained by the partial reduction of this catalytic system during OSR-LNG reaction conditions. The phase composition studies performed for the 20% Ni/La_2_O_3_ (D), 20% Ni/5% CeO_2_–La_2_O_3_ (E) and 20% Ni/5% ZrO_2_–La_2_O_3_ (F) catalysts are presented in Figure 6. The XRD curves recorded for the D, E and F catalysts after the calcination process confirmed the rather amorphous state of these samples. The diffraction peaks which were visible in Figure 6 can be assigned to nickel, cerium, zirconium and lanthanum (regular and hexagonal structure) oxides and LaNiO_3_ phases, but also La_2_O_2_NO_3_, La_2_O_2_CO_3_ and La(OH)_3_ phases, which are the remainder of the precursor and were not decomposed during the calcination process [45]. Additionally, the metallic Ni phase was detected on the diffraction curves recorded for the spent nickel catalysts supported on La_2_O_3_ (D), 5% CeO_2_–La_2_O_3_ (E) and 5% ZrO_2_–La_2_O_3_ (F) systems. The diffraction peaks attributed to NiO phases were visible in the case of the 20% Ni/5% CeO_2_–La_2_O_3_ (E) and 20% Ni/5% ZrO_2_–La_2_O_3_ (F) catalysts. The detailed analysis of the XRD patterns (Figure 6) also showed the occurrence of the diffraction peaks attributed to La_2_O_3_ hex., La_2_O_3_ reg., ZrO_2_ and CeO_2_ phases. The XRD studies conducted by another research group [48] concentrated on La_2_O_3_ and Ni/La_2_O_3_ catalyst systems calcined in the temperature range 450 °C–850 °C showed peaks on the X-ray patterns assigned to NiO (positioned at 2θ angles of 42.9° and 62.8°) and LaNiO_3_ (positioned at 2θ angles of 23.3° and 32.9°) phases. The authors also reported that calcination at 450 °C does not completely convert lanthanum hydroxide to lanthanum oxide [49]. In addition, lanthanum oxide could react with water and carbon dioxide, which are present in air, forming the La(OH)_3_ and La_2_O_2_CO_3_ compounds [50]. The chemical reactions presented below show these processes:

La_2_O_3_ + H_2_O → La(OH)_3_

The average size of the nickel oxide and metallic nickel crystallites for the calcined and spent nickel catalysts were calculated based on Scherrer’s formula [27]. The results of the calculation are given in Table 3. The crystallite size of the NiO species for calcined nickel catalysts supported on CeO_2_, 5% La_2_O_3_–CeO_2_ and 5% ZrO_2_–CeO_2_ were in the range of 26–59 nm, whereas the Ni catalysts supported on the La_2_O_3_, 5% CeO_2_–La_2_O_3_ and 5% ZrO_2_–La_2_O_3_ systems showed lower NiO crystallite sizes, which equaled about 5 nm. These results are connected with the amorphous state of the investigated catalytic systems. The obtained results clearly showed that in all cases after thevoxy-steam reforming of LNG reaction, the metallic Ni crystallites were larger compared to the NiO crystallite sizes of the nickel catalysts after the calcination process. Moreover, the largest Ni and NiO crystallite size was observed for the 20% Ni/5% ZrO_2_–CeO_2_ catalyst. It is also worth mentioning that the introduction of CeO_2_ or ZrO_2_ to La_2_O_3_ oxide resulted in a decrease in the size of the metallic nickel crystallites, with 67 nm detected for 20%Ni/La_2_O_3_ to 29 and 41 nm for the 20% Ni/5% CeO_2_–La_2_O_3_ and 20% Ni/5% ZrO_2_–La_2_O_3_ spent catalysts in the OSRM of LNG process. In the case of the Ni catalysts supported on 5% ZrO_2_–CeO_2_ or 5% La_2_O_3_–CeO_2_ carriers, the XRD results showed the opposite trend. These spent Ni catalysts (20%Ni/5% ZrO_2_–CeO_2_—153 nm, 20%Ni/5% La_2_O_3_–CeO_2_—47 nm) exhibited higher metallic nickel crystallite sizes compared to the 20% Ni/CeO_2_ system (37 nm). These behaviors may also explain the reactivity of the investigated catalysts in the studied reaction. Based on the obtained results and the activity tests it can be concluded that Ni systems characterized by the higher crystallite sizes of metallic Ni on the catalyst surface exhibited higher activity in the studied process. On the other hand, for the calcined Ni catalysts supported on La_2_O_3_, 5% CeO_2_–La_2_O_3_ and 5% ZrO_2_–La_2_O_3_, we confirmed practically the same sizes of the NiO crystallites on their surface, which were equal to 5, 6 and 4 nm, respectively. In contrast, the calcined Ni catalyst supported on 5% ZrO_2_–CeO_2_ exhibited an NiO crystallite size of 59 nm and this result confirms that the modification of the CeO_2_ support by means of ZrO_2_ increases the nickel oxide crystallite size. However, in the case of the La_2_O_3_ oxide the addition of this modifier resulted in a decrease in the nickel oxide crystallite size from 28 nm (which was detected for 20%Ni/CeO_2_ system) to 26 nm (for the 20%Ni/5% La_2_O_3_-CeO_2_ catalyst).

### 2.4. Reduction Behavior of Ni Catalysts

**Figure 7:**
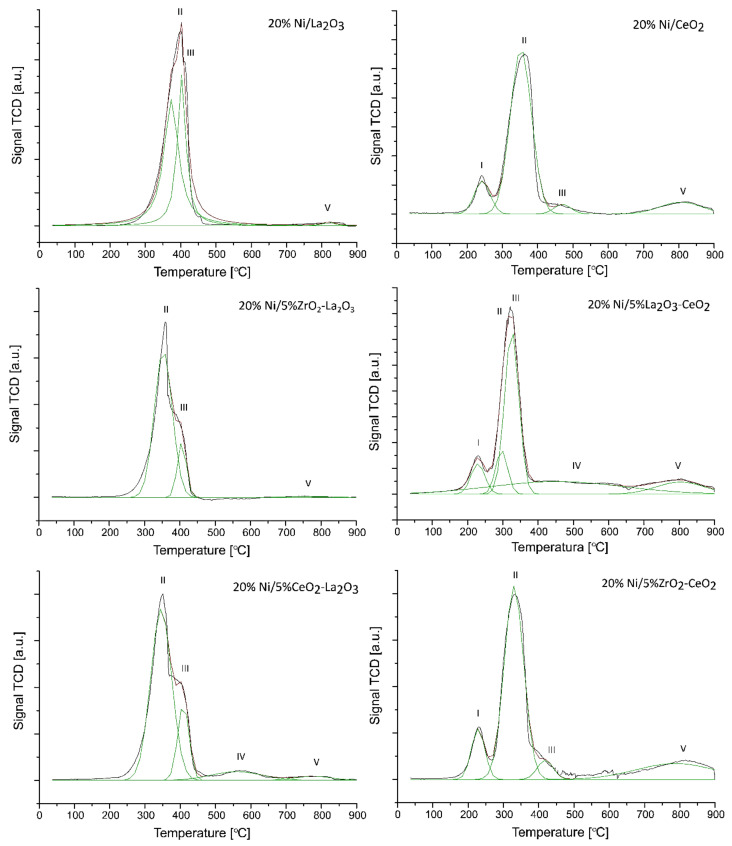
TPR-H_2_ profiles of nickel catalysts calcined at 400 °C in an air atmosphere for 4 h.

The reduction behavior of the investigated supported nickel catalysts were determined using the temperature-programmed reduction technique (TPR-H_2_). TPR-H_2_ studies were performed to explain the interactions between the active phase and support components of the investigated nickel catalysts. The TPR-H_2_ profiles recorded for the studied catalyst systems are shown in Figure 7. The TPR-H_2_ curve recorded for the 20% Ni/La_2_O_3_ catalyst showed three reduction stages, with the maximum of H_2_ consumption at about 400 °C, 410 °C and 850 °C, respectively. The first two reduction effects are related to the reduction of free and NiO species interacting with the support [45]. The high temperature reduction effect located at about 850 °C is assigned to the reduction of the NiLaO_3_ compound [51]. The TPR-H_2_ profiles recorded for 20% Ni/5% CeO_2_–La_2_O_3_ and 20% Ni/5% ZrO_2_–La_2_O_3_ catalysts showed the same reduction effects as those observed for 20% Ni/La_2_O_3_ but shifted towards a lower temperature range. This result proved that the addition of CeO_2_ and ZrO_2_ to 20% Ni/La_2_O_3_ catalysts facilitates NiO species reduction. In the case of the 20% Ni/5% ZrO_2_–La_2_O_3_ catalyst, we also observed three reduction stages, assigned to the same oxidic phase reduction as the 20% Ni/La_2_O_3_ system. On the other hand, the TPR profile of the 20% Ni/5% CeO_2_–La_2_O_3_ catalyst showed four reduction effects. The maximum value of H_2_ consumption for these reduction stages are located at about 350 °C, 385 °C, 575 °C and 810 °C, respectively. The presented reduction peaks are assigned to the reduction of free NiO, NiO species interacting with the support, Ce oxy-species present on the catalyst surface [52] and the NiLaO_3_ compound, respectively. Yingzhi Yu et al. [48] investigated the Ni/La_2_O_3_ catalysts calcined in the temperature range of 450 °C–850 °C. The H_2_-TPR results recorded for these catalysts showed three reduction peaks. The first (300 °C–500 °C) and second (500 °C–600 °C) reduction stages are attributed to NiO weakly and strongly interacting with the carrier, whereas the last reduction profile (>600 °C) was assigned to the spinel and perovskite LaNiO_3_ compound reduction, which confirmed the strong interaction between Ni^2+^ and the support [53,54]. Moreover, Maria Martha Barroso-Quiroga and Adolfo Eduardo Castro-Luna [55] also studied the reduction behavior of Ni/La_2_O_3_ catalysts. The TPR profile recorded for this catalyst showed three reduction steps. The first reduction peak with the maximum of H_2_ consumption at 565 K is related with NiO reduction and the last two peaks, positioned at about 690 and 791 K, are assigned to the reduction of La_2_NiO_4_ oxide, the presence of which was confirmed by means of the XRD technique. As shown in the same Figure, the TPR-H_2_ profile of the 20% Ni/CeO_2_ system showed four reduction stages (marked with numbers I, II, III and V). The first two effects located at 245 °C and 365 °C are connected with the reduction of free NiO and NiO interacting with the support, respectively. The next two reduction stages, located at about 465 °C and 825 °C, are associated with the reduction of Ce-oxy species presented on the catalyst surface and the bulk CeO_2_ reduction to non-stochiometric CeO_2-x_ species. The results also showed that the addition of 5% La_2_O_3_ and 5% ZrO_2_ oxides to the Ni/CeO_2_ system improves the NiO reduction, which is manifested by the shift of the reduction effects towards a lower temperature range in comparison to the 20% Ni/CeO_2_ catalyst. The reduction effects shown on the TPR curves recorded for the 20% Ni/5% La_2_O_3_–CeO_2_ and 20% Ni/5% ZrO_2_–CeO_2_ catalysts correspond to the reduction stages for the nickel catalyst supported on CeO_2_. The reduction behavior of NiO species depends on their size and their interaction with the support components [56,57]. It is well known that NiO strongly interacting with a cerium support will be reduced at a higher temperature range [58]. Greluk et al. [59] reported that the reduction effects recorded for the Ni/CeO_2_ catalyst in the temperature range 220 °C–450 °C are related to the reduction of NiO weakly and strongly interacting with the cerium oxide carrier. They also attributed these peaks to the surface reduction of nonstoichiometric CeO_x_ forms. The authors observed a reduction peak at a high temperature above 600 °C, which was associated with the reduction of bulk CeO_2_ to Ce_2_O_3_ oxide [60,61].

To confirm the promoting effect of the addition of La_2_O_3_, CeO_2_ or ZrO_2_ oxide on the reduction of nickel oxide species, deconvolution calculations were performed and the results are presented in Table 4. The contribution of each peak to the total area under the TPR peaks can be attributed to the reduction of various forms of NiO and support components. Table 4 also presents the maximum of each of the reduction peaks. The deconvolution results clearly show that the addition of ZrO_2_ to a CeO_2_ support in the case of nickel catalysts decreases the intensity of the peaks attributable to the reduction of NiO interacting with the support (peak II), at the same time increasing the intensity of the peaks attributed to the reduction of Ce-oxy species (peak III) and resulting in the bulk reduction of CeO_2_ to non-stochiometric CeO_2-x_ (V-peak). We observed the same trend in the case of the Ni catalysts supported on the 5% La_2_O_3_–CeO_2_ system. In addition, the intensity of the reduction peaks observed on the TPR profile for this catalyst, which were assigned to the reduction of free NiO and NiO species interacting with the support (peaks I–III), decreased in parallel with the increasing intensity of the reduction peak attributed to Ce-oxy species reduction (peak IV). Moreover, the addition of CeO_2_ and ZrO_2_ to the La_2_O_3_ support of the Ni catalyst caused an increase in the intensity of the free NiO reduction peak (peak II) and a decrease in the reduction peak attributed to NiO interacting with the catalyst support (peak III). The shift of the reduction effects on TPR profiles towards the lower temperature range proves that the addition of CeO_2_, La_2_O_3_ or ZrO_2_ oxides facilitates the reduction of different nickel species present on the catalyst surface. This reduction behavior has a significant influence on the reactivity results observed in the oxy–steam reforming of LNG.

### 2.5. FTIR Analysis

**Figure 8:**
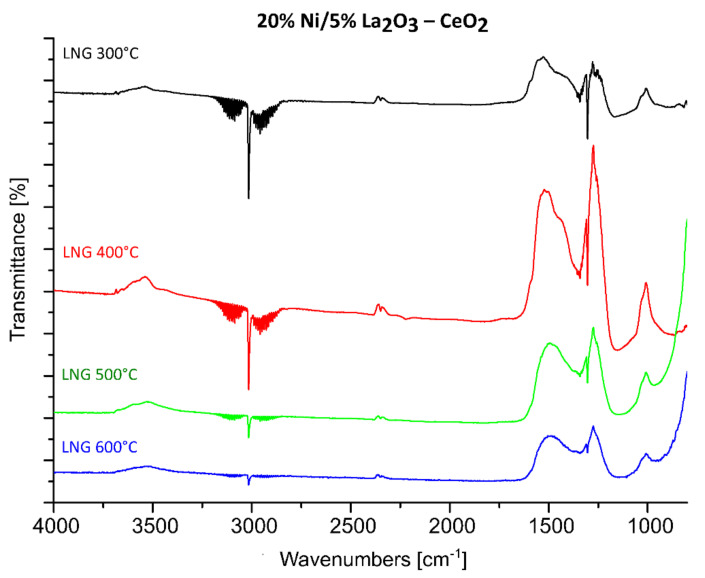
In situ IR spectra under LNG decomposition process conditions in the temperature range 300 °C–600 °C recorded for 20% Ni/5% La_2_O_3_–CeO_2_ catalyst.

**Figure 9:**
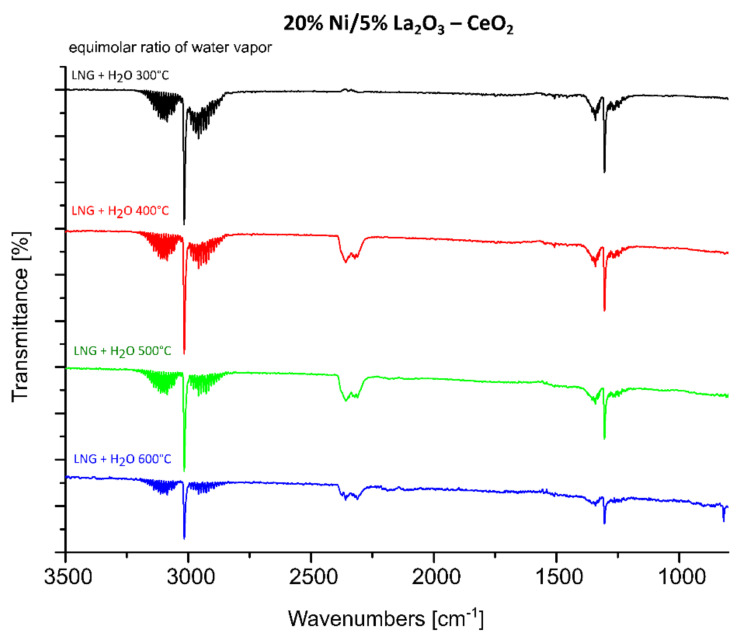
In situ IR spectra under steam reforming of LNG conditions in the temperature range 300 °C–600 °C recorded for the 20% Ni/5% La_2_O_3_–CeO_2_ catalyst.

**Figure 10:**
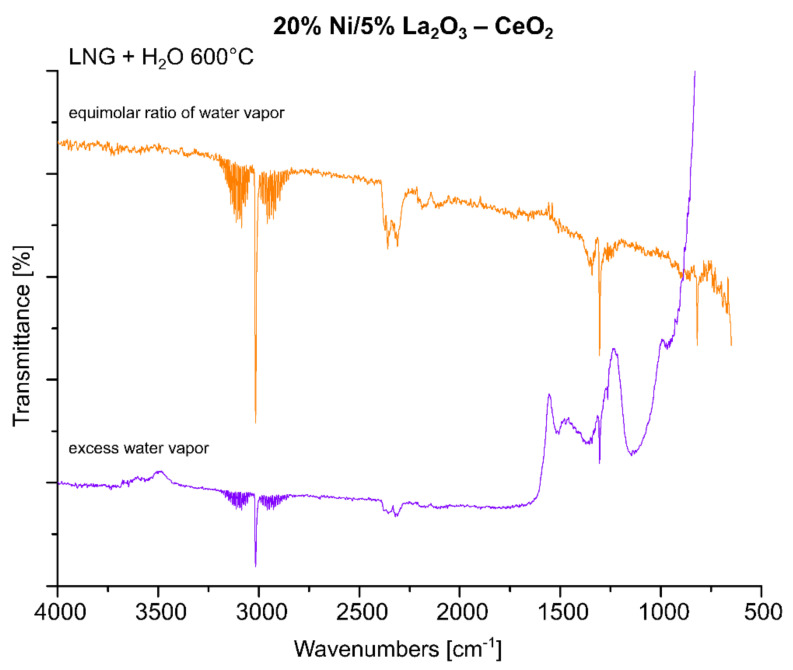
In situ IR spectra under steam reforming of LNG conditions at 600 °C with equimolar and excess water vapor recorded for the 20% Ni/5% La_2_O_3_–CeO_2_ catalyst.

**Figure 11:**
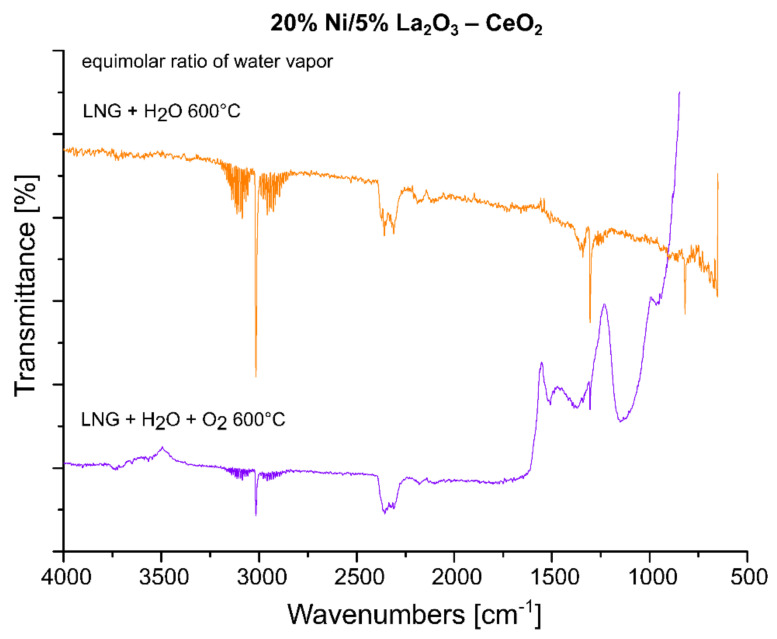
In situ IR spectra under steam and oxy-steam reforming of LNG conditions at 600 °C with equimolar water vapor, recorded for the 20% Ni/5% La_2_O_3_–CeO_2_ catalyst.

**Figure 12:**
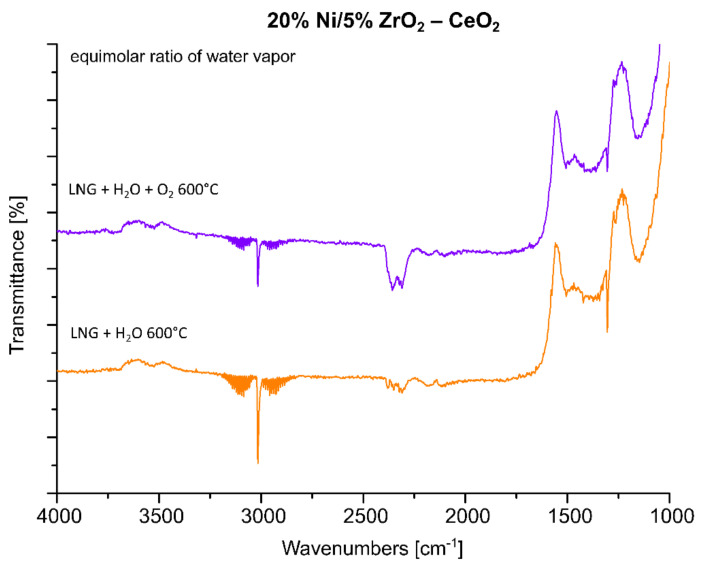
In situ IR spectra under steam and oxy-steam reforming of LNG conditions at 600 °C recorded for the 20% Ni/5% ZrO_2_–CeO_2_ catalyst.

**Figure 13:**
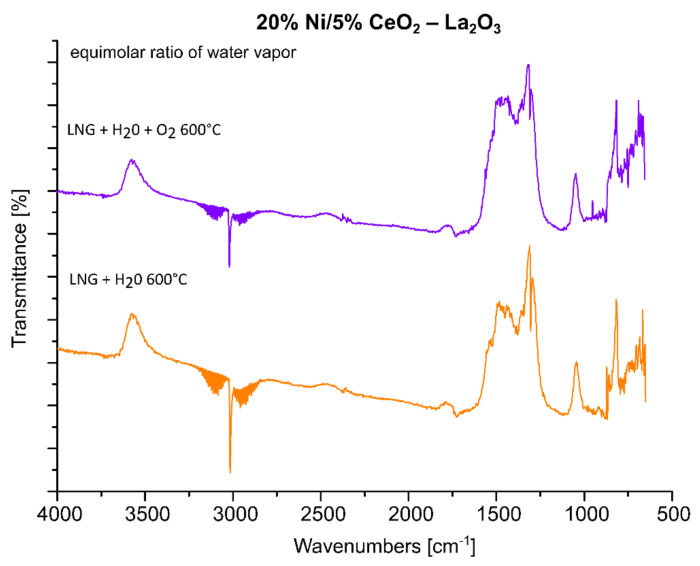
In situ IR spectra under steam and oxy-steam reforming of LNG conditions at 600 °C recorded for the 20% Ni/5% CeO_2_–La_2_O_3_ catalyst.

FTIR measurements were performed in order to understand the catalytic activity and OSR-LNG reaction mechanism of the studied nickel-supported catalysts. Three selected catalytic systems were extensively studied using the FTIR technique. FTIR measurements were carried out for the most active 20% Ni/5% La_2_O_3_–CeO_2_ and 20% Ni/5% ZrO_2_–CeO_2_ systems, along with the least active one 20% Ni/5% CeO_2_–La_2_O_3_. The IR spectra of the adsorbed species formed on the catalysts’ surfaces during the LNG decomposition, the steam reforming of LNG (SR-LNG) and the oxy–steam reforming of LNG (OSR-LNG) processes were analyzed in the temperature range of 300 °C–600 °C. The obtained results are given in Figure 8, Figure 9, Figure 10, Figure 11, Figure 12 and Figure 13, respectively. Figure 8 shows the IR spectra obtained for the 20% Ni/5% La_2_O_3_–CeO_2_ catalyst collected during LNG decomposition process conditions in the temperature range 300 °C–600 °C. The IR spectra showed several characteristic bands located at the wavenumbers in the range 2800–3200 cm^−1^ and 800–1650 cm^−1^.

The bands located in the range 2800–3200 cm^−1^ are assigned to the components of the LNG mixture. In addition, the sharp band visible on the FTIR spectra positioned at about 1300 cm^−1^ is also connected with the LNG components. The results of our studies showed that in the temperature range from 300 °C to 600 °C the decomposition of the LNG on the investigated catalyst takes place to a limited extent. This result agrees well with those of other works [62], in which only a low conversion value of methane was observed up to 500 °C. The FTIR band located in the wavenumbers ranging from 1200 to 1600 cm^−1^ are assigned to the presence of different carbonate species formed during the decomposition reaction from the CO_2_ via the Boudouard reaction [63]. The bands located between 800 and 1100 cm^−1^ can be attributed to the other carbon compound, containing a C-C bond. Figure 9 presents the FTIR studies performed for the same catalytic systems tested in the process of the steam reforming of LNG in the temperature range of 300 °C–600 °C. The FTIR spectra collected for the same catalyst, 20% Ni/5% La_2_O_3_–CeO_2_, in the steam reforming of LNG also showed the specific bands assigned to LNG components (2800–3200 cm^−1^ and at 1300 cm^−1^). In addition, in Figure 9 the corresponding bands attributed to the reaction product CO_2_ (2305, 2350 cm^−1^) are visible on the collected spectra, starting from the temperature of 400 °C. Furthermore, CO can be observed on the FTIR spectra (2115 and 2172 cm^−1^), starting from 500 °C. These results agree well with our activity tests for the studied material. Namely, carbon dioxide was observed as a reaction product starting from 300 °C as a result of the water–gas shift reaction, and carbon monoxide from a temperature of 500 °C, from which temperature the steam methane reforming and the partial oxidation of methane reactions began to occur. These results confirm that at a low temperature in the steam reforming of LNG, the water–gas shift reaction is the major process which occurs during the ongoing process, as evidenced by the reaction products. The increase in the reaction temperature to 500 °C results in appearance of CO in the final product, which confirms that at high temperatures, steam methane reforming of LNG is favored, as evidenced by the increased intensity of bands assigned to the reaction products of CO and CO_2_. In addition, at 500 °C the increase in the specific bands assigned to gaseous CO was observed, which confirms that at a higher temperature, the steam reforming reaction and the partial oxidation of the hydrocarbons included in the LNG are favored.

In the next step of the FTIR measurements, the steam reforming of LNG was realized on the same system with equimolar and excess amounts of water at 600 °C. The results of the IR measurements were presented in Figure 10. One can see in the presented data that the increase in the water amount during the process results in a decrease in the intensity of the specific bands attributed to gaseous CO_2_, CO and LNG components. The spectra obtained also confirm the appearance of bands attributed to carbonate forms resulting from the decomposition of carbon dioxide (1220–1600 cm^−1^) and to carbon compounds containing a C-C bond (800–1100 cm^−1^). In addition, small bands located on the IR spectrum around 3400 cm^−1^ during the process are assigned to the OH stretching frequency. These bands can also be attributed to the adsorption of water molecules produced during the process or to excess water introduced from the reaction mixture. The obtained results, presented in Figure 10, confirmed the higher efficiency of the process carried out using the reaction mixture containing excess water vapor, as evidenced by the high intensity of the specific bands attributed to carbonate groups formed on the IR spectra during the tested process and the low intensity of IR bands assigned to LNG components. In the next step, the FTIR measurements were performed for the same catalytic material in the oxy-steam reforming of LNG. The IR spectra collected for the investigated material in the steam and oxy-steam reforming of LNG are shown in Figure 11. The presented results show clearly that the introduction of O_2_ into the reaction mixture resulted in increases in the characteristic bands assigned to gaseous CO_2_ and a decrease in the gaseous CO. In addition, the catalytic tests performed for this system showed that, starting from 600 °C, higher conversion of LNG components was observed compared to the process performed at lower temperatures. This also means that in the presence of oxygen in the reaction mixture, a higher content of CO_2_ is formed from the oxidation of the decomposition products of hydrocarbons.

Analogical measurements were also performed for the 20% Ni/5% ZrO_2_–CeO_2_ catalyst, which also showed high activity during the oxy-steam reforming of LNG process. The FTIR spectra collected for this catalytic system showed the same tendency which was detected for the 20% Ni/5% La_2_O_3_–CeO_2_ system (see Figure 12). These results confirmed our observation that the introduction of oxygen into the mixture of LNG components and steam results in an increase in the conversion of LNG components and the intensity of the IR bands assigned to gaseous carbon dioxide, which decomposes to the main product, CO. For a comparison, analogous measurements were also performed for the least active catalyst. The results of the FTIR measurements are presented in Figure 13. The FTIR results also confirmed the low activity of this system at 600 °C in the oxy-steam reforming of LNG. The results agree well with the catalytic activity and also indicate that at 600 °C gaseous CO was not formed during the process and only gaseous CO_2_ and carbonate species adsorbed on the catalyst surface were observed. The catalytic activity results obtained for this catalyst clearly show that up to 700 °C, we only detected CO_2_ in the product stream (see the activity results). Increasing the temperature of the process up to 900 °C resulted in the appearance of the reaction products CO and H_2_. This means that only at this temperature did the reforming of the LNG components take place in the 20% Ni/5% CeO_2_–La_2_O_3_ and 20% Ni/5% ZrO_2_–La_2_O_3_ catalyst systems. These results may be explained by the strong acidity of those systems compared to the most active catalysts, which exhibited lower values of total acidity. The least active catalysts probably show strong sorption properties in relation to carbon monoxide, which in the next stage linked to the catalyst surface was oxidized to carbon dioxide. These results were confirmed by a large number of medium and strong acid sites present on the catalyst surface.

Based on the catalytic activity and FTIR results, we can propose the mechanism of the oxy-steam reforming of LNG. Firstly, at low temperatures, only CO_2_ was observed in the effluent gas as a reaction product. At low temperatures, oxygen and water vapor from the reaction mixture may react with the surface nickel atoms, generating surface oxygen and hydroxyl groups. These species may take a part in the oxidation process of radicals chemisorbed on the surface, mainly with CH_x_ forming from the decomposition of the hydrocarbon components of LNG. The oxidation process of the CH_x_ species results in carbon dioxide formation. It is also worth mentioning that at low temperatures of the process CO_2_ was observed as the only reaction product for the least active catalyst system. This means that CO_2_ is formed via the WGS reaction or is formed through the oxidation process of CH_x_ species chemisorbed on the surface. Oxygen or hydroxyl species present on the catalyst surface or oxygen from the reaction mixture may also interact with carbon-containing radicals and form CH_2_O, CHO, CO or even CO_2_. Increasing the reaction temperature up to 500 °C results in the appearance of CO and hydrogen products in the most active catalytic systems. Furthermore, increasing the reaction process above 600 °C caused CO_2_ to be detected as a minor product. The thermodynamics of the process makes the steam reforming process or partial oxidation of the hydrocarbons contained in the liquefied natural gas a privileged reaction. The main products of the oxy-steam reforming of LNG are CO and hydrogen. The hydrogen formed is directly released into the gas phase and/or the atomic hydrogen exists as an adsorbed species on the catalyst surface. However, in the case of the least active systems, it is postulated that the carbon monoxide formed in the reaction is strongly bound by the surface of the catalytic system, thus preventing desorption as a reaction product, and it is at this stage oxidized to carbon dioxide. Only at a high temperature of 900 °C is the easy desorption of the carbon monoxide formed under the reaction conditions possible, which was confirmed by the results in regard to catalytic activity.

### 2.6. SEM-EDS Analysis

**Figure 14:**
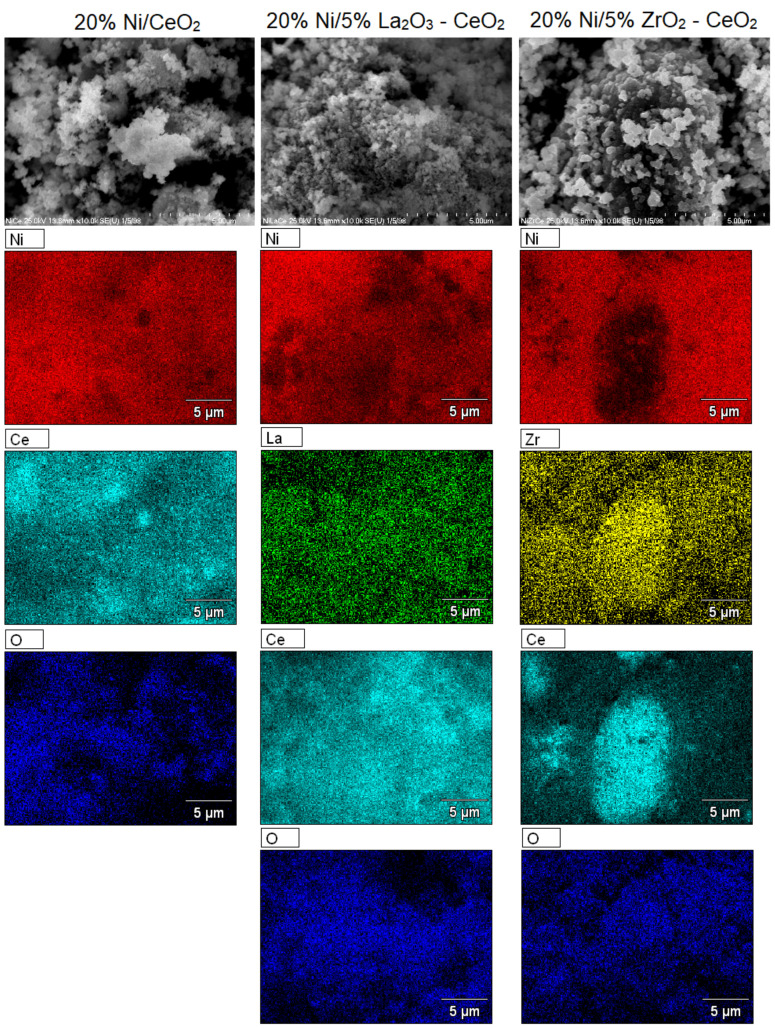
SEM images of 20% Ni/CeO_2_, 20% Ni/5% La_2_O_3_–CeO_2_ and 20% Ni/5% ZrO_2_–CeO_2_ catalysts calcined in air atmosphere at 400 °C for 4 h.

**Figure 15:**
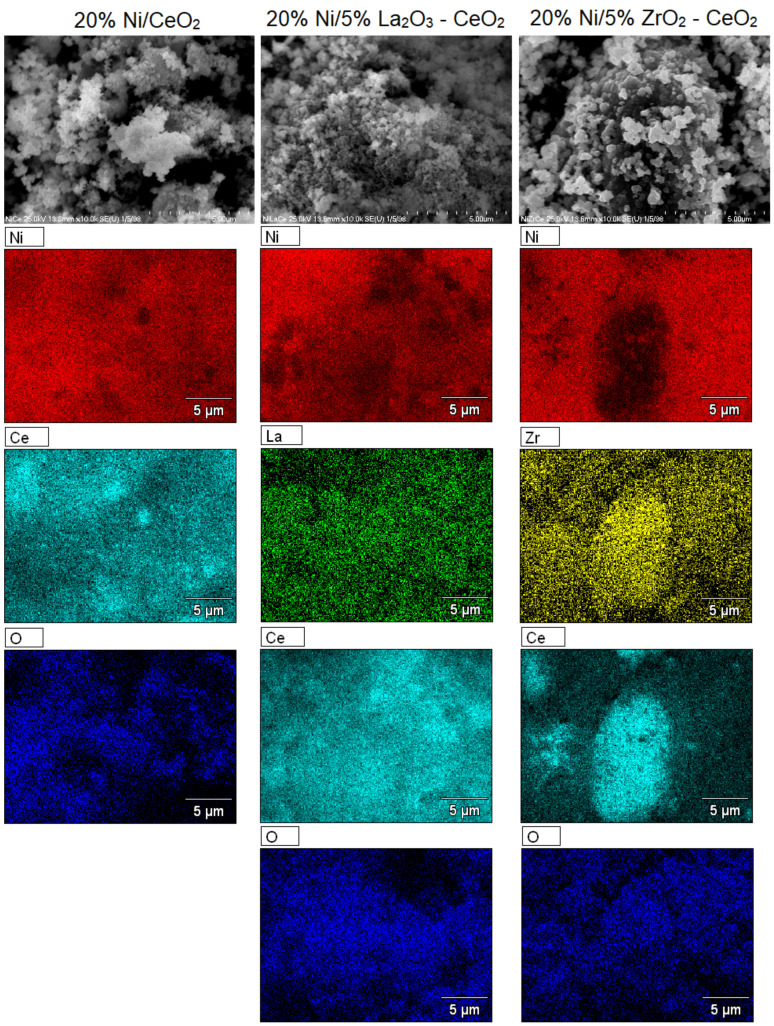
SEM images of 20% Ni/La_2_O_3_, 20% Ni/5% CeO_2_–La_2_O_3_ and 20% Ni/5% ZrO_2_–La_2_O_3_ catalysts calcined in air atmosphere at 400 °C for 4 h.

SEM-EDS measurements were performed for nickel-supported catalysts after calcination performed at 400 °C in an air atmosphere for 4 h. Scanning electron microscopy with an energy dispersive detector was used to determine the morphology and composition of the investigated catalyst surfaces. SEM images collected for the studied catalysts are presented in Figure 14 and Figure 15. The presented data clearly confirm the composition of the supported nickel catalysts tested. Nickel was detected on all studied catalyst surfaces. The presence of elements such as Ce, Ni and O was confirmed on the EDS spectra recorded for all Ni catalysts supported on CeO_2_, 5% La_2_O_3_–CeO_2_ and 5% ZrO_2_–CeO_2_. On the EDS spectra recorded for all Ni catalysts supported on La_2_O_3_, 5% CeO_2_–La_2_O_3_ and 5% ZrO_2_–La_2_O_3_ systems the presence of the elements Ni, La and O was detected. In addition, for the 20% Ni/5% La_2_O_3_–CeO_2_ and 20%Ni/5% ZrO_2_–CeO_2_ catalysts we also detected the elements La and Zr on their surface, respectively. The surface regions with nickel, lanthanum and cerium in the case of the 20% Ni/5% La_2_O_3_–CeO_2_ and 20% Ni/5% CeO_2_–La_2_O_3_ catalysts and nickel, zirconium and cerium in the case of a 20% Ni/5% ZrO_2_–CeO_2_ catalysts can be easily distinguished. On the other hand, regions with nickel, zirconium and lanthanum were also detected in the case of the 20% Ni/5% ZrO_2_–La_2_O_3_ catalyst. These results confirm the presence of specific interactions between the catalyst components, which can influence on their reactivity in the oxy-steam reforming of LNG. SEM images recorded for the investigated catalysts showed different distributions of elements on their surfaces. The lack of other elements on the catalysts’ surfaces proves that other impurities were not introduced during the catalyst preparation process, which could affect the catalytic activity of Ni systems in the oxy-steam reforming of LNG.

### 2.7. Catalytic Performance

**Figure 16:**
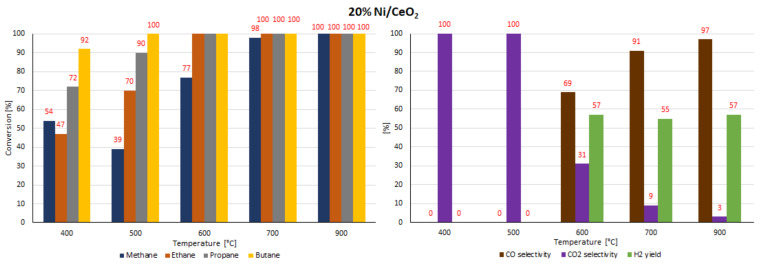
The activity results of the 20% Ni/CeO_2_ catalyst in the OSR-LNG process.

**Figure 17:**
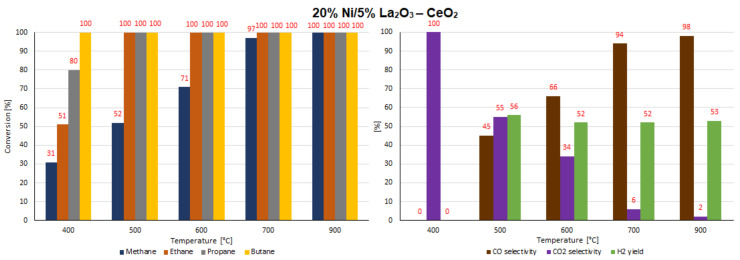
The activity results of the 20% Ni/5% La_2_O_3_–CeO_2_ catalyst in the OSR-LNG process.

**Figure 18:**
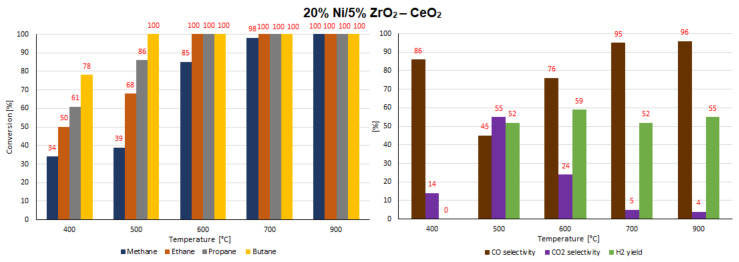
The activity results of the 20% Ni/5% ZrO_2_–CeO_2_ catalyst in the OSR-LNG process.

**Figure 19:**
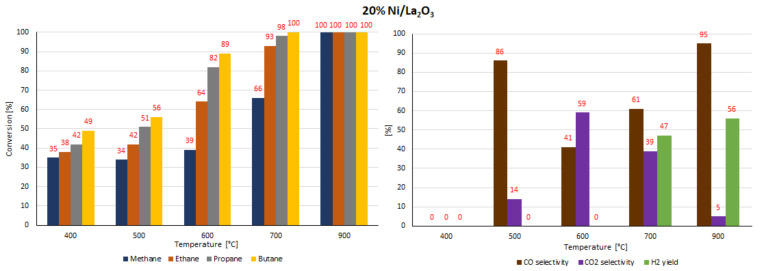
The activity results of the 20% Ni/La_2_O_3_ catalyst in the OSR-LNG process.

**Figure 20:**
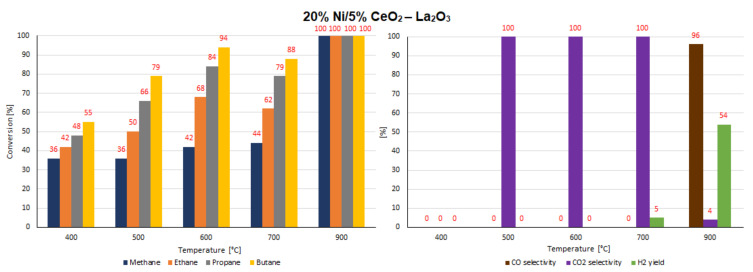
The activity results of the 20% Ni/5% CeO_2_–La_2_O_3_ catalyst in the OSR-LNG process.

**Figure 21:**
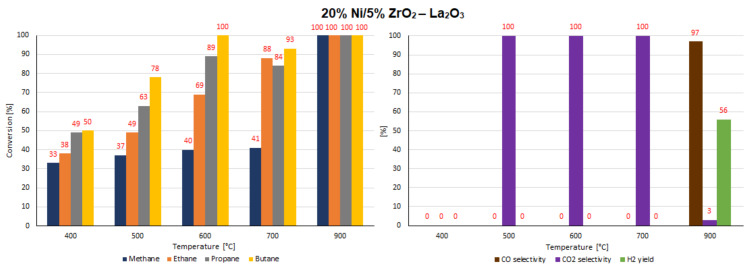
The activity results of the 20% Ni/5% ZrO_2_–La_2_O_3_ catalyst in the OSR-LNG process.

The catalytic activity tests of all investigated supported nickel catalysts were performed in the oxy-steam reforming of LNG (OSR-LNG) process. The catalytic activity measurements were carried out in a fixed bed microreactor under atmospheric pressure in the temperature range of 400 °C–900 °C. The main aim of the activity tests was to determine the influence of the addition of CeO_2_, ZrO_2_ and La_2_O_3_ oxides on the LNG conversion and syngas production of nickel catalysts supported on CeO_2_ and La_2_O_3_ systems. The catalytic activity was expressed in terms of LNG component conversion, selectivity to CO and CO_2_, and H_2_ yield for all investigated catalyst systems, and these results are presented in Table 5 and Table 6 and Figure 16, Figure 17, Figure 18, Figure 19, Figure 20 and Figure 21, respectively. The most active catalyst in the tested reaction performed at 500 °C was the 20% Ni/5% La_2_O_3_–CeO_2_ system, which already showed full ethane, propane and butane conversion and a high hydrogen yield (56%) at 500 °C. On the other hand, the highest active catalyst in OSR-LNG at 600 °C was the 20% Ni/5% ZrO_2_–CeO_2_ system, which showed the highest CH_4_ (85%) and full C_2_H_6_, C_3_H_8_ and C_4_H_10_ conversion. Moreover, this catalyst also showed the highest H_2_ yield, at 59%. The nickel catalysts supported on La_2_O_3_, 5% CeO_2_–La_2_O_3_ and 5% ZrO_2_–La_2_O_3_ systems demonstrated significant lower activity in the studied OSR-LNG reaction compared to nickel catalysts based on CeO_2_ supports. These catalytic systems reached the complete conversion of higher hydrocarbons (ethane, propane and butane) at the highest reaction temperature (900 °C). The hydrogen appeared in the product of the reaction at 700 °C and 900 °C for 20% Ni/La_2_O_3_ and 20% Ni/5% CeO_2_–La_2_O_3_ catalysts and at 900 °C for the 20% Ni/5% ZrO_2_–La_2_O_3_ systems. Furthermore, for the nickel catalysts supported on La_2_O_3_, 5% CeO_2_–La_2_O_3_ and 5% ZrO_2_–La_2_O_3_ systems at 400 °C we did not observe H_2_ and CO formation. The catalytic activity measurements performed for Ni catalysts supported on 5%CeO_2_–La_2_O_3_ and 5%ZrO_2_–La_2_O_3_ systems showed that during the oxy-steam reforming of LNG carried out in the temperature range from 500 °C to 700 °C, only CO_2_ was formed as a reaction product. It is worth mentioning that the increase in the temperature of the OSR-LNG reaction results in the appearance, apart from CO_2_, of CO among the reaction products (see Table 6 and Figure 19, Figure 20 and Figure 21).

**Figure 22:**
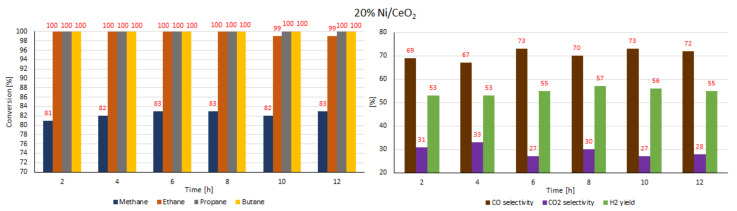
Results of the time-on-stream catalytic tests performed over 12 h of the OSR of LNG reaction on a 20% Ni/CeO_2_ catalyst.

**Figure 23:**
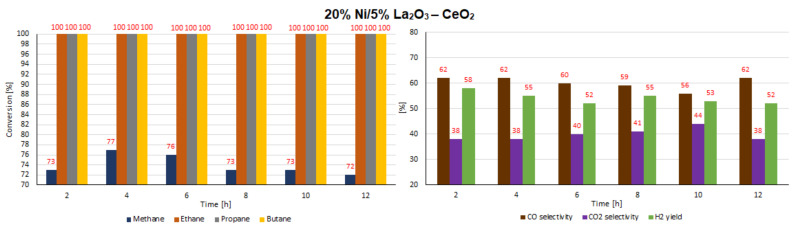
Results of the time-on-stream catalytic tests performed over 12 h of the OSR of LNG reaction on a 20% Ni/5% La_2_O_3_–CeO_2_ catalyst.

**Figure 24:**
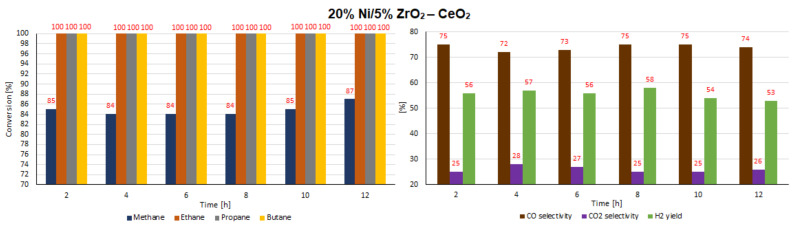
Results of the time-on-stream catalytic tests performed over 12 h of the OSR of LNG reaction on a 20% Ni/5% ZrO_2_–CeO_2_ catalyst.

In the next step of the catalytic activity measurements, the stability tests were carried out over 12 h at 600 °C in the oxy-steam reforming of LNG for the three most active catalysts (20% Ni/CeO_2_, 20% Ni/5% La_2_O_3_–CeO_2_ and 20% Ni/5% ZrO_2_–CeO_2_). The results of the stability tests performed during the oxy-steam reforming of LNG are presented in Table 7 and Table 8 and Figure 22, Figure 23 and Figure 24, respectively. The results of the time-on-stream catalytic tests showed that all the studied catalysts were stable during this process. The 20% Ni/CeO_2_ catalyst exhibited stable activity during the 10 h of the running the process. It showed stable CH_4_ conversion, ranging from 81%–83%. However, after 10 h on the stream, the catalyst had not yet achieved the full conversion of ethane. The conversion was 99%. The stability testes showed also that the CO and CO_2_ concentrations fluctuated in the ranges of 69%–73% and 27%–33%, respectively. It should be emphasized that the 20% Ni/CeO_2_ catalyst had reached the highest value of H_2_ yield, at 57%, after only 8 h of the process. The 20% Ni/CeO_2_ catalyst with the addition of ZrO_2_ exhibited stable methane conversion and carbon oxide concentrations (CO and CO_2_) during the running process. The hydrogen yield fluctuated in range of 52%–58% and the highest value, 58%, was reached after 8 h of stability tests. The last tested catalyst (20% Ni/5% La_2_O_3_–CeO_2_) showed a slight increase in methane conversion values in the 6-h catalytic tests to 76% and after that we observed a slight decrease in this value to 72%. The H_2_ yield after 2 h of the test stabilized at the highest value of 58% and then decreased to 52%. The CO and CO_2_ concentrations fluctuated in the ranges of 56%–62% and 38%–44%, respectively.

## 3. Materials and Methods

### 3.1. Supports and Catalysts Preparation

CeO_2_ and La_2_O_3_ mono-oxide supports were synthesized using the precipitation method. The precipitation process was carried out using an ammonia solution as a precipitation agent until the pH reached the value of 10. In the next step, the cerium or lanthanum hydroxides were filtrated, washed and dried in an air atmosphere at 120 °C for 2 h. Finally, the CeO_2_ and La_2_O_3_ carriers were calcined for 4 h at 400 °C and 700 °C, respectively. The CeO_2_ support modified by 5% La_2_O_3_ or 5% ZrO_2_ and the La_2_O_3_ support modified by 5% CeO_2_ or 5% ZrO_2_ were prepared via the impregnation method. The impregnation process lasted 12 h, then the catalytic materials were evaporated, dried for 2 h at 80 °C and calcined in an air atmosphere for 4 h at 400 °C for 5% La_2_O_3_–CeO_2_ and 5% ZrO_2_–CeO_2_ supports and at 700 °C in the case of 5% CeO_2_–La_2_O_3_ and 5% ZrO_2_–La_2_O_3_ supports. Monometallic nickel catalysts were also prepared using the conventional impregnation method. NiO oxide was introduced onto the support surfaces using a nickel (II) nitrate hexahydrate precursor. The impregnation process took 12 h. The catalytic materials were then dried for 2 h at 80 °C and calcined in an air atmosphere for 4 h at 400 °C, in all cases. The Ni loading in the case of the investigated catalysts was 20 wt.%.

### 3.2. Catalysts Characterization

The specific surface area and porosity of the investigated monometallic nickel catalysts were determined using the BET method. The analyses were carried out in an ASAP 2020 Micrometrics apparatus (Surface Area and Porosity Analyzer, Micromeritics Instrument Corporation, Norcross, GA, USA). The distributions of the pore sizes were determined using the BJH method. The acidic properties of the catalytic materials were studied via the temperature programmed desorption of ammonia technique (TPD-NH_3_). In each experiment, the catalyst samples were reduced at 500 °C in a mixture of 5% H_2_–95% Ar and the TCD detector monitored the concentration of ammonia in the temperature range of 100 °C–600 °C (homemade apparatus). The reducibility of the nickel catalysts were investigated via the temperature programmed reduction technique (TPR-H_2_), using an automatic AMI-1 instrument (Altamira Instruments, Pittsburgh, PA, USA). The reduction behaviors of the catalytic materials were determined in the temperature range of 35 °C–900 °C. The phase compositions of the synthesized materials were determined using the X-ray diffraction technique in a PANalytical X’Pert Pro MPD diffractometer (Malvern Panalytical Ltd., Royston, UK) in Bragg–Brentano reflection geometry. The morphology and distribution of the elements on the catalyst surfaces were studied using a S-4700 scanning electron microscope from HITACHI (S-4700 HITACHI, Tokyo, Japan), equipped with EDX detector (ThermoNoran, Madison, WI, USA). Infrared spectra were recorded with a Nicolet iS50 FT-IR spectrometer (Thermo Scientific, Waltham, MA, USA) equipped with a liquid-nitrogen-cooled MCT detector. A resolution of 4.0 cm^−1^ was used throughout the investigation. Sixty-four scans were taken to achieve a satisfactory signal-to-noise ratio. The background spectrum was collected before each measurement in the selected temperature range.

### 3.3. Catalytic Activity and Stability Measurements

The catalytic activity and stability tests of the studied supported nickel catalysts were carried out during the oxy-steam reforming of liquefied natural gas reaction (OSR-LNG). The LNG mixture contains hydrocarbons such as methane (5%), ethane (0.4%), propane (0.2%) and butane (0.05%). The catalytic tests were performed in a quartz micro-reactor in the temperature range of 400 °C–900 °C under atmospheric pressure. The weight of a catalyst sample was 0.2 g in all cases. In each catalytic test, the activities were measured after 30 min of the stabilization process in the reaction mixture. The stability tests were performed for the selected catalysts at 600 °C for 12 h. The stability results were collected every 2 h. The total gas flow rate of the reaction mixture was 51 cm^3^/min and argon was used as a balance gas. The molar ratio between each component in the reaction mixture was C:H_2_O:O_2_ = 1:2.7:0.35. The analysis of the gaseous products before and after the OSR–LNG reaction was carried out using gas chromatographs equipped with TCD and FID detectors. The catalytic activity results were expressed as the conversion of hydrocarbons (methane, ethane, propane, butane), selectivity to CO and CO_2_ and hydrogen yield, which were calculated based on the following Equations (1)–(4):

(1) $$C_{X}H_{Y}{}_{Conv.\;\;}=\;\left(1-\;\frac{n-out{\;}_{C_{x}H_{Y}}}{n-in{\;}_{C_{x}H_{Y}}}\right)\times 100\;\%$$

(2) $$CO_{Sel.}\;=\left(\frac{n-out{\;}_{CO}}{n-out_{\;CO}\;+\;n-out{\;}_{CO_{2}}}\right)\;\times 100\;\%$$

(3) $$CO_{2}{}_{Sel.}\;=\left(\frac{n-out{\;}_{CO_{2}}}{n-out_{\;CO}\;+\;n-out{\;}_{CO}{}_{2}}\right)\;\times 100\;\%$$

(4) H2Yield = n−out H22.73∑n−in CxHY−  ∑n−out CxHY ×100 %

where:

$n-in{\;}_{C_{x}H_{Y}}$—the moles of hydrocarbon (methane, ethane, propane and butane) at the reactor inlet;
$n-out{\;}_{C_{x}H_{Y}}$—the moles of hydrocarbon (methane, ethane, propane and butane) at the reactor outlet;
$n-out{\;}_{CO}$—the moles of CO at the reactor outlet;
$n-out{\;}_{CO}{}_{2}$—the moles of CO_2_ at the reactor outlet;
$n-out{\;}_{H_{2}}$—the moles of H_2_ at the reactor outlet;
$\sum \left(n-in{\;}_{C_{x}H_{Y}}\right){\;}_{\;}$—the sum of the moles of the hydrocarbons (methane, ethane, propane and butane) at the reactor inlet;
$\sum \left(n-out{\;}_{C_{x}H_{Y}}\right)$—the sum of the moles of the hydrocarbons (methane, ethane, propane and butane) at the reactor outlet.

## 4. Conclusions

In the present study, monometallic nickel catalysts supported on mono CeO_2_, La_2_O_3_ and the binary oxides 5% ZrO_2_–CeO_2_, 5% CeO_2_–La_2_O_3_ and 5% ZrO_2_–La_2_O_3_ were prepared via the impregnation method and tested in the oxy-steam reforming of LNG. The results confirm the possibility of carrying out the oxy-steam reforming of LNG at 600 °C on prepared nickel catalysts. A description of the mechanism of the oxy-steam reforming of LNG was proposed based on the FTIR and activity results. The highest activity in the studied process at 500 °C was shown by 20% Ni/5% CeO_2_–La_2_O_3_ catalysts, which also demonstrated high stability in the investigated process. The 20% Ni/5% La_2_O_3_–CeO_2_ system exhibited full ethane, propane and butane conversion and a high hydrogen yield (56%) at 500 °C, whereas, the most active catalyst in the OSR-LNG process at 600 °C was the 20% Ni/5% ZrO_2_–CeO_2_ system, which showed the highest CH_4_ (85%) and full C_2_H_6_, C_3_H_8_ and C_4_H_10_ conversion. Moreover, this catalyst also showed the highest H_2_ yield, at 59%. The least active systems were the catalysts with a higher content of La_2_O_3_ oxide. The low activity of these catalysts may be explained by the formation of LaNiO_3_ compounds during the calcination step. The presence of this compound was confirmed via the XRD and TPR-H_2_ techniques. However, when it comes to highly active nickel catalysts, their activity is explained by their relatively higher specific surface area compared to catalysts containing a higher La content. For these systems, hardly reducible compounds consisting of active phase and carrier components (e.g., NiLaO_3_) were not observed. In addition, the intermediate products of the process were less bound on their surfaces, which caused an increase in catalytic activity.

## Figures and Tables

**Table 1:** The specific surface area, monolayer capacity and average pore size for monometallic supported nickel catalysts calcined in an air atmosphere at 400 °C for 4 h.

Materials	BET Surface Area (m^2^/g)	Monolayer Capacity (cm^3^/g)	Average Pore Radius (nm)
CeO_2_	20.08	0.10	9.76
5% ZrO_2_–CeO_2_	30.53	0.10	5.41
5% La_2_O_3_–CeO_2_	19.39	0.09	6.89
La_2_O_3_	0.44	0.002	19.93
5% CeO_2_–La_2_O_3_	4.91	0.03	14.82
5% ZrO_2_–La_2_O_3_	3.27	0.02	16.53
20% Ni/CeO_2_	16.69	0.09	9.98
20% Ni/5% ZrO_2_–CeO_2_	15.93	0.08	7.92
20% Ni/5% La_2_O_3_–CeO_2_	16.98	0.09	10.36
20% Ni/La_2_O_3_	4.28	0.03	12.70
20% Ni/5% CeO_2_–La_2_O_3_	12.07	0.06	9.57
20% Ni/5% ZrO_2_–La_2_O_3_	6.74	0.04	11.40

**Table 2:** The amount of NH_3_ adsorbed on the surface of monometallic supported nickel catalysts reduced at 500 °C in pure hydrogen for 1 h, calculated from the surface under the peaks recorded during the TPD-NH_3_ measurements.

	Total Acidity (mmol/g)	Weak Centers (mmol/g)	Medium Centers (mmol/g)	Strong Centers (mmol/g)
	180 °C–600 °C	180 °C–300 °C	300 °C–450 °C	450 °C–600 °C
20% Ni/CeO_2_	0.30	0.22	0.05	0.03
20% Ni/5% ZrO_2_–CeO_2_	0.47	0.13	0.21	0.13
20% Ni/5% La_2_O_3_–CeO_2_	0.22	0.03	0.07	0.12
20% Ni/La_2_O_3_	0.35	0.02	0.21	0.12
20% Ni/5% CeO_2_–La_2_O_3_	1.69	0.01	0.31	1.37
20% Ni/5% ZrO_2_–La_2_O_3_	0.70	0.05	0.30	0.35

**Table 3:** The sizes of the NiO and metallic Ni crystallites, calculated based on the XRD measurements of calcined and spent Ni catalysts in the OSR of LNG.

Catalysts	The Size of NiO Crystallites (nm)	The Size of Metallic Ni Crystallites (nm)
20% Ni/CeO_2_	28	37
20% Ni/5% ZrO_2_–CeO_2_	59	153
20% Ni/5% La_2_O_3_–CeO_2_	26	47
20% Ni/La_2_O_3_	5	67
20% Ni/5% CeO_2_–La_2_O_3_	6	29
20% Ni/5% ZrO_2_–La_2_O_3_	4	41

**Table 4:** Reduction data for the nickel-supported catalysts after calcination in an air atmosphere for 4 h at 400 °C.

Catalysts	Peak Contribution to the Overall TPR Peak Area (%)				
	I-Peak (T_max_)	II-Peak (T_max_)	III-Peak (T_max_)	IV-Peak (T_max_)	V-Peak (T_max_)
20% Ni/CeO_2_	0.11 (245 °C)	0.75 (365 °C)	0.03 (465 °C)	-	0.11 (825 °C)
20% Ni/5% ZrO_2_–CeO_2_	0.11 (230 °C)	0.63 (340 °C)	0.05 (400 °C)	-	0.21 (830 °C)
20% Ni/5% La_2_O_3_–CeO_2_	0.08 (230 °C)	0.09 (310 °C)	0.42 (320 °C)	0.30 (500 °C)	0.11 (800 °C)
20% Ni/La_2_O_3_	-	0.35 (400 °C)	0.62 (410 °C)	-	0.03 (850 °C)
20% Ni/5% CeO_2_–La_2_O_3_	-	0.71 (350 °C)	0.17 (385 °C)	0.09 (575 °C)	0.03 (810 °C)
20% Ni/5% ZrO_2_–La_2_O_3_	-	0.83 (360 °C)	0.15 (390 °C)	-	0.02 (780 °C)

**Table 5:** Conversion values of the hydrocarbons obtained in the process of the oxy-steam reforming of liquefied natural gas (LNG) using supported nickel catalysts.

Catalysts	Temp (°C)	Methane Conversion (%)	Ethane Conversion (%)	Propane Conversion (%)	Butane Conversion (%)
20% Ni/CeO_2_	400	37	47	72	92
	500	39	70	90	100
	600	77	100	100	100
	700	98	100	100	100
	900	100	100	100	100
20% Ni/5% ZrO_2_–CeO_2_	400	34	50	61	78
	500	39	68	86	100
	600	85	100	100	100
	700	98	100	100	100
	900	100	100	100	100
20% Ni/5% La_2_O_3_–CeO_2_	400	31	51	80	100
	500	52	100	100	100
	600	71	100	100	100
	700	97	100	100	100
	900	100	100	100	100
20% Ni/La_2_O_3_	400	35	38	42	49
	500	34	42	51	56
	600	39	64	82	89
	700	66	93	98	100
	900	100	100	100	100
20% Ni/5% CeO_2_–La_2_O_3_	400	36	42	48	55
	500	36	50	66	79
	600	42	68	84	94
	700	44	62	79	88
	900	100	100	100	100
20% Ni/5% ZrO_2_–La_2_O_3_	400	33	38	49	50
	500	37	49	63	78
	600	40	69	89	100
	700	41	88	84	93
	900	100	100	100	100

**Table 6:** The hydrogen yield and selectivity values to CO and CO_2_ obtained in the oxy-steam reforming of LNG using supported nickel catalysts.

Catalysts	Temp (°C)	CO Selectivity (%)	CO_2_ Selectivity (%)	H_2_ Yield (%)
20% Ni/CeO_2_	400	0	100	0
	500	0	100	0
	600	69	31	57
	700	91	9	55
	900	97	3	57
20% Ni/5% ZrO_2_–CeO_2_	400	86	14	0
	500	45	55	52
	600	76	24	59
	700	95	5	52
	900	96	4	55
20% Ni/5% La_2_O_3_–CeO_2_	400	0	100	0
	500	45	55	56
	600	66	34	52
	700	94	6	52
	900	98	2	53
20% Ni/La_2_O_3_	400	0	100	0
	500	86	14	0
	600	41	59	0
	700	61	39	47
	900	95	5	56
20% Ni/5% CeO_2_–La_2_O_3_	400	0	100	0
	500	0	100	0
	600	0	100	0
	700	0	100	5
	900	96	4	54
20% Ni/5% ZrO_2_–La_2_O_3_	400	0	100	0
	500	0	100	0
	600	0	100	0
	700	0	100	0
	900	97	3	56

**Table 7:** Conversion values of the hydrocarbons obtained in the stability tests in the oxy-steam reforming of liquefied natural gas (LNG), performed over 12 h time-on-stream using supported nickel catalysts.

Catalysts	Time (h)	Methane Conversion (%)	Ethane Conversion (%)	Propane Conversion (%)	Butane Conversion (%)
20% Ni/CeO_2_	2	81	100	100	100
	4	82	100	100	100
	6	83	100	100	100
	8	83	100	100	100
	10	82	99	100	100
	12	83	99	100	100
20% Ni/5% ZrO_2_–CeO_2_	2	85	100	100	100
	4	84	100	100	100
	6	84	100	100	100
	8	84	100	100	100
	10	85	100	100	100
	12	87	100	100	100
20% Ni/5% La_2_O_3_–CeO_2_	2	73	100	100	100
	4	77	100	100	100
	6	76	100	100	100
	8	73	100	100	100
	10	73	100	100	100
	12	72	100	100	100

**Table 8:** The hydrogen yield and selectivity values to CO and CO_2_ obtained in the stability tests in the oxy-steam reforming of liquefied natural gas (LNG) performed over 12 h time-on-stream using supported nickel catalysts.

Catalysts	Time (h)	CO Selectivity (%)	CO_2_ Selectivity (%)	H_2_ Yield (%)
20% Ni/CeO_2_	2	69	31	53
	4	67	33	53
	6	73	27	55
	8	70	30	57
	10	73	27	56
	12	72	28	55
20% Ni/5% ZrO_2_–CeO_2_	2	75	25	56
	4	72	28	57
	6	73	27	56
	8	75	25	58
	10	75	25	54
	12	74	26	53
20% Ni/5% La_2_O_3_–CeO_2_	2	62	38	58
	4	62	38	55
	6	60	40	52
	8	59	41	55
	10	56	44	53
	12	62	38	52

## Data Availability

The results presented in this work were not previously published anywhere.
